# Frequency of Three-Vessel Disease Among Patients With Non-ST Segment Elevation Myocardial Infarction

**DOI:** 10.7759/cureus.11634

**Published:** 2020-11-22

**Authors:** Faisal Ahmed, Muhammad Sami Khan, Syed Dilbahar Ali Shah, Javed Jalbani, Arshad Ali Shah, Ghulam Abbas Shaikh

**Affiliations:** 1 Cardiology, Dow University of Health Sciences, Civil Hospital Karachi, Karachi, PAK; 2 Internal Medicine, Dr. Ruth K. M. Pfau Civil Hospital Karachi, Karachi, PAK; 3 Interventional Cardiology, National Institute of Cardiovascular Diseases, Karachi, PAK; 4 Cardiology, Dr. Ruth K. M. Pfau Civil Hospital Karachi, Karachi, PAK

**Keywords:** three vessel disease, non-st-segment elevation myocardial infarction, angiography, coronary artery occlusion

## Abstract

Background: Though the presence of three-vessel disease (3VD) among patients with non-ST Segment Elevation Myocardial Infarction (NSTEMI) is relatively common, very limited data is available regarding its clinical significance. The current study aimed to determine the frequency of 3VD among NSTEMI patients presenting at the tertiary care hospital of Karachi, Pakistan.

Methodology: This cross-sectional study was conducted at the National Institute of Cardiovascular Diseases, Karachi from August 15 2015 to February 15 2016 over a sample of 139 NSTEMI patients. Data regarding patients' baseline characteristics were recorded in a proforma. Coronary angiography was performed to determine the presence of 3VD. The frequency of occluded arteries and 3VD was also recorded and the collected data was then analyzed using Statistical Package for Social Sciences (SPSS) version 20.0 (IBM Corp., Armonk, NY, USA).

Results: A total of 139 NSTEMI patients were enrolled in the study with a mean age of 50.47 ± 12.47 years. The majority of them were males (70.5%), mostly ≥ 40 years of age (67.6%). Among the comorbidities, 50.4% of patients had diabetes mellitus (DM), 61.9% were hypertensive and 30.9% had dyslipidemia. The overall frequency of 3VD among the enrolled NSTEMI patients was 30.2%. Three major arteries were found to be occluded; 68.3% NSTEMI patients had occlusion in the left anterior descending (LAD) artery, followed by right coronary artery (RCA) among 49.6%, 40.3% had left circumflex (LCX) artery occlusion, and 50.4% had diagonal and obtuse marginal (OM) artery occlusion respectively. Among the effect modifiers, no significant impact of age, gender, and smoking habits was observed on the frequency of 3VD among NSTEMI patients (p > 0.05). Among the reported comorbidities, DM was significant among NSTEMI patients with 3VD (p < 0.05).

Conclusion: Our study results indicated that a significant proportion of NSTEMI patients had 3VD, independent of the effect of age and gender.

## Introduction

Coronary artery disease (CAD) with its massive frequency has become a major public health concern and is now among the leading causes of morbidity and mortality worldwide [1-2]. It is estimated that annually 17.9 million deaths are reported concerning cardiovascular diseases and of these 7.4 million are related to CAD, as per the reports issued by the World Health Organization (WHO) [1]. CAD mostly remains asymptomatic while a sub-category, acute coronary syndrome (ACS) is always symptomatic and has a frequent association with myocardial infarction (MI). The ACS further sub-divides as ST Segment Elevation Myocardial Infarction (STEMI) and Non-ST Segment Elevation Myocardial Infarction (NSTEMI) which is relatively more common and is one of the major reasons behind ACS-associated hospital admissions and deaths [3-4]. 

Both STEMI and NSTEMI are characterized by occlusion of one or more culprit arteries, but the condition is known to be less severe among NSTEMI patients, which may be due to the incomplete occlusion of the culprit arteries as compared to STEMI cases where arteries undergo complete occlusion [5-6]. As per the existing literature, the angiographic features and the rate of occluded arteries among NSTEMI patients are nearly 25% similar to those of STEMI patients [5-6]. Additionally, patients with NSTEMI with occluded arteries (NSTEMIOA) display a higher mortality rate and are more prone to heart failure as compared to NSTEMI with a patent artery (NSTEMIPA) [5-7]. Furthermore, the presence of three-vessel disease (3VD) among ACS patients is considered the most severe and fatal form of CAD, as it occludes three major coronary arteries including the Left Anterior Descending (LAD) artery, Right Coronary Artery (RCA) and Left Circumflex (LCX) artery, carrying high morbidity and mortality risk [8-9]. It has been widely observed among patients with either stable or unstable coronary artery disease [9], and such patients are considered endangered and difficult to manage as per the therapeutic guidelines [10].

Coronary angiography is performed in the routine clinical practice among both STEMI and NSTEMI patients in order to identify the occluded area of the coronary artery [11]. The European Society of Cardiology (ESC) Guidelines suggest that patients with either STEMI or unstable UNSTEMI must undergo angiography within less than two hours of index hospitalization while a delay of 24 to 48 hours can only be considered in case of stable NSTEMI [12]. The angiographic and re-vascularization delay causes an irreversible negative impact on myocardial functioning [6]. However, the presence of 3VD among NSTEMI patients further adds prognostic complexities which can lead to poor long-term process [13], which can be improved by percutaneous or surgical revascularization [14]. As per the clinical guidelines, invasive strategies with cardiac catheterization, revascularization, and initiation of dual antiplatelet therapy is recommended for treatment among NSTEMI patients with 3VD [15].

To date, limited data is available determining the frequency of 3VD among patients with NSTEMI in our population. Therefore, the current study was aimed to estimate the frequency of 3VD among NSTEMI patients presenting at the National Institute of Cardiovascular Diseases, Karachi, Pakistan.

## Materials and methods

This cross-sectional study was conducted at the Medical Wards of National Institute of Cardiovascular Diseases, Karachi for six months from August 15 2015 to February 15 2016. A sample size of 139 was calculated through non-probability consecutive sampling technique, using the online website www.openepi.com (version 3.01), taking the prevalence of 3VD among patients with NSTEMI as 23% [16], confidence interval at 95%, and margin of error at 7%. All the patients with diagnosed NSTEMI, aged > 18 and < 70 years and provided consent to participate were included in the study. Admitted patients with diagnosed unstable angina/STEMI, fibrinolysis, cardiogenic shock, pregnant females, and non-consenting participants were excluded from the study sample.

The study was ethically approved by the Ethical Review Committee of the College of Physicians & Surgeons, Pakistan, and the National Institute of Cardiovascular Diseases (NICVD). Written informed consent was taken before enrollment in the study. Baseline demographic details including age, gender, comorbidities, smoking status, and dyslipidemia were recorded in a proforma. The frequency of occluded or culprit arteries was also noted. All the patients underwent coronary angiography by experienced consultant cardiologists practicing for more than three years. Among the outcome variable, the frequency of 3VD among the enrolled NSTEMI patients was observed.

The data were analyzed using Statistical Package for Social Sciences (SPSS) version 20.0 (IBM Corp., Armonk, NY, USA), the mean and standard deviation was calculated for age. While for categorical variables like gender, diabetes mellitus (DM), hypertension (HTN), smoking, dyslipidemia, involved arteries, and occurrence of 3VD, frequency along with percentage was given. To observe the effect of baseline demographics on the outcome variable i.e. 3VD occurrence rate, the Chi-square test was used where p-value ≤ 0.05 was considered significant.

## Results

A total of 139 patients fulfilling the inclusion criteria were enrolled in the study, with a mean age of 50.47 ± 12.47 years having 67.63% ≥ 40 years of age. The sample presented a male majority i.e. 70.5% of the enrolled patients were males. The details regarding comorbidities, smoking status, presence of dyslipidemia, and involved arteries are presented in Table 1. 

**Table 1:** Baseline characteristics of the study population *Values are given as n(%) or mean ± SD

Variables		(n=139)
Age (Years)		50.47 ± 12.47
Age groups	< 40 years	45(32.37)
	≥ 40 years	94(67.63)
Gender	Male	98(70.50)
	Female	41(29.50)
Diabetes Mellitus		70(50.4)
Hypertension		86(61.9)
Smoking		66(47.5)
Dyslipidemia		43(30.9)

The distribution of occluded arteries was also noted as shown in Table 2. Of them, LAD was found to be involved in most cases (68.3%), followed by diagonal branches and obtuse marginal (OM) artery. Significant 3VD was observed among 30.22% of the NSTEMI patients.

**Table 2:** Frequency and distribution of occluded coronary arteries and outcome variables. *LAD- left anterior descending; RCA- right coronary artery; LCX- left circumflex artery; OM- Obtuse marginal artery

Variables		n(%)
Occluded Arteries	LAD	95(68.3)
	RCA	69(49.6)
	LCX	56(40.3)
	Diagonal Branches	70(50.4)
	OM	70(50.4)
Significant Three Vessel Disease	Present	42(30.2)
	Absent	97(69.7)

Although no significant association was found between age and 3VD occurrence, it was observed that 3VD was more frequent among patients ≥ 40 years of age and males. Among the comorbid conditions, DM was found to have a significant effect on the 3VD frequency (p = 0.000) (Table 3).

**Table 3:** Frequency of Three Vessel Disease for baseline characteristics and occluded arteries. *p-value < 0.05 is considered significant. *LAD- left anterior descending; RCA- right coronary artery; LCX- left circumflex artery; OM- Obtuse marginal artery

Variables		Three Vessel Disease		p-value
		Present n (%)	Absent n (%)	
Age groups	< 40 years	17(40.4)	28(28.8)	0.126
	≥ 40 years	25(59.5)	69(71.1)	
Gender	Male	32(76.1)	66(68.0)	0.224
	Female	10(23.8)	31(31.9)	
Diabetes Mellitus		38(90.4)	32(33.0)	<0.001*
Hypertension		14(33.3)	72(74.2)	<0.001*
Smoking		22(52.4)	44(45.4)	0.282
Dyslipidemia		15(35.7)	28(28.9)	0.272
Occluded Arteries	LAD	39(92.8)	56(57.7)	<0.001*
	RCA	22(52.4)	47(48.5)	0.405
	LCX	21(50)	35(36.1)	0.089
	Diagonal	22(52.4)	48(49.5)	0.449
	OM	24(57.1)	46(47.4)	0.193

## Discussion

The outcomes, severity, and mortality among the NSTEMI patients completely depend on the intensity of the myocardial injury, diagnosis, management, and co-existing disease conditions. The incidence of coronary artery occlusion among NSTEMI patients is frequent and has long been known. It is evident from the existing literature that older age, male gender, and higher resting heart rate are independent positive predictors of the presence of 3VD among NSTEMI patients [13]. In the current study involving 139 NSTEMI patients, 42 (30.2%) had significant 3VD. Moreover, the incidence was more frequent among males (76.1%) and in the older age group (59.5%). Although similar to our findings, the 3VD incidence was high among males but contrastingly profound in the younger age group [17].

Furthermore, NSTEMI patients with a comorbid condition like DM showed a high frequency of 3VD (p < 0.001). Similarly, a study assessing the prevalence, clinical outcomes, and predictors of left main (LM) and/or 3VD in NSTEMI patients, indicated that the majority of the NSTEMI patients with LM/3VD were diabetic or hypertensive (p < 0.001) [13]. Additionally, the occlusion was fairly distributed among the coronary arteries, i.e. 92.8% of the enrolled NSTEMI patients with 3VD had significant occlusion in the LAD artery followed by 57.1% in OM artery, 52.4% in both RCA and diagonal arteries and 50% in LCX artery, however, a study found that NSTEMI patients with 3VD were more likely to have an unidentified culprit artery (p < 0.001) [13].

In addition to age, gender, and comorbidities, the effect of smoking on the frequency of 3VD was also observed. Smoking has been significantly associated with CAD [18]. But no significant association was found between smoking habit and the presence of 3VD (p = 0.282) in our study. On the contrary, a study indicated that NSTEMI patients, usually young smokers with diabetes, are more likely to have 3VD [19].

There are certain limitations in our study that should be considered while interpreting these findings. It was a single-center study and the data was limited to a small subset of the population. The risk profile, mortality, or associated adversities were not assessed. Descriptive long-term follow-up studies focusing on the in-hospital stay, mortality, and complications among the local population affected with NSTEMI, will probably add to the existing data.

## Conclusions

It is concluded from the study results that the patients with NSTEMI are likely to have 3VD. Although the incidence is more frequent among male patients of ≥ 40 years of age, no significant effect of age, gender, and smoking status were observed on the reported frequency of 3VD among NSTEMI patients. Moreover, a significant proportion of these patients had DM.

## References

[1] (2017). Cardiovascular Diseases (CVDs). January.

[2] Khan R, Akhter J, Munir U, Almas T (2020). Frequency of non-ST segment elevation myocardial infarction (NSTEMI) In acute coronary syndrome with normal electrocardiogram (ECG): insights from a cardiology hospital in Pakistan. Cureus.

[3] Ruff CT, Braunwald E (2011). The evolving epidemiology of acute coronary syndromes. Nat Rev Cardiol.

[4] Basit H, Malik A, Huecker MR (2019). Non ST Segment Elevation (NSTEMI) Myocardial Infarction. https://www.ncbi.nlm.nih.gov/books/NBK513228/.

[5] Soon K, Du HN, Klim S, Zakariyya A, Kelly A-M (2014). Non-ST elevation myocardial infarction with occluded artery and its clinical implications. Heart Lung Circ.

[6] Amsterdam EA, Wenger NK, Brindis RG (2014). 2014 AHA/ACC guideline for the management of patients with non-ST-elevation acute coronary syndromes: a report of the American College of Cardiology/American Heart Association task force on practice guidelines. J Am Coll Cardiol.

[7] Shin DI (2014). Impact of occluded culprit arteries on long-term clinical outcome in patients with non-ST-elevation myocardial infarction: 48-month follow-up results in the COREA-AMI registry. J Interv Cardiol.

[8] Levine GN, Bates ER, Blankenship JC (2012). 2011 ACCF/AHA/SCAI guideline for percutaneous coronary intervention: executive summary: a report of the American College of Cardiology Foundation/American Heart Association Task Force on Practice Guidelines and the Society for Cardiovascular Angiography and Interventions. Catheter Cardiovasc Interv.

[9] D'Ascenzo F, Presutti DG, Picardi E (2012). Prevalence and non-invasive predictors of left main or three-vessel coronary disease: evidence from a collaborative international meta-analysis including 22 740 patients. Heart.

[10] Baumann AA, Mishra A, Worthley MI, Nelson AJ, Psaltis PJ (2020). Management of multivessel coronary artery disease in patients with non-ST-elevation myocardial infarction: a complex path to precision medicine. Ther Adv Chronic Dis.

[11] Roe MT, White JA, Kaul P (2012). Regional patterns of use of a medical management strategy for patients with non-ST-segment elevation acute coronary syndromes: insights from the EARLY ACS trial. Circ Cardiovasc Qual Outcomes.

[12] Roffi M, Patrono C, Collet J-P (2016). 2015 ESC Guidelines for the management of acute coronary syndromes in patients presenting without persistent ST-segment elevation: Task Force for the Management of Acute Coronary Syndromes in Patients Presenting without Persistent ST-Segment Elevation of the European Society of Cardiology (ESC). Eur Heart J.

[13] Carvalho JF, Belo A, Congo K (2018). Left main and/or three-vessel disease in patients with non-ST-segment elevation myocardial infarction and low-risk GRACE score: prevalence, clinical outcomes and predictors. Rev Port Cardiol.

[14] Mohr FW, Morice M-C, Kappetein AP (2013). Coronary artery bypass graft surgery versus percutaneous coronary intervention in patients with three-vessel disease and left main coronary disease: 5-year follow-up of the randomised, clinical SYNTAX trial. Lancet.

[15] Ahrens I, Averkov O, Zúñiga EC (2019). Invasive and antiplatelet treatment of patients with non‐ST‐segment elevation myocardial infarction: understanding and addressing the global risk‐treatment paradox. Clin Cardiol.

[16] Kouchoukos N, Blackstone E, Doty D, Hanley F, Karp R (2003). Cardiac surgery. Anatomy, Dimensions and Terminology.

[17] Yousaf H, Saad AA, Bhutta MI, Babar HA, Ahmed N, Mohyudin MT (2012). Frequency of significant three vessel coronary artery disease in non st-segment elevation myocardial infarction having low high density lipoprotein level. Pak Heart J.

[18] Islam KN, Chowdhury AW, Khandaker AH, Sabah KM, Amin MG, Kabir SR, Saleh MA (2015). Comparison of different risk factors and coronary angiographic profile in younger and older patients with ischemic heart disease. Cardiovascular J.

[19] Sharma R, Bhairappa S, Prasad SR, Manjunath CN (2014). Clinical characteristics, angiographic profile and in hospital mortality in acute coronary syndrome patients in south Indian population. Heart India.

